# Epstein-Barr Virus and Its Association with Oral Hairy Leukoplakia: A Short Review

**DOI:** 10.1155/2016/4941783

**Published:** 2016-03-07

**Authors:** Razia Abdool Gafaar Khammissa, Jeanine Fourie, Rakesh Chandran, Johan Lemmer, Liviu Feller

**Affiliations:** Department of Periodontology and Oral Medicine, Sefako Makgatho Health Sciences University, Ga-Rankuwa 0208, South Africa

## Abstract

In immunocompromised subjects, Epstein-Barr virus (EBV) infection of terminally differentiated oral keratinocytes may result in subclinical productive infection of the virus in the stratum spinosum and in the stratum granulosum with shedding of infectious virions into the oral fluid in the desquamating cells. In a minority of cases this productive infection with dysregulation of the cell cycle of terminally differentiated epithelial cells may manifest as oral hairy leukoplakia. This is a white, hyperkeratotic, benign lesion of low morbidity, affecting primarily the lateral border of the tongue. Factors that determine whether productive EBV replication within the oral epithelium will cause oral hairy leukoplakia include the fitness of local immune responses, the profile of EBV gene expression, and local environmental factors.

## 1. Introduction

**Figure 1:**
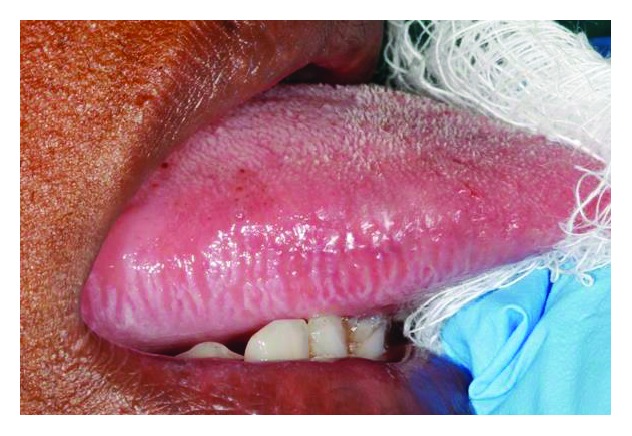
Mild oral hairy leukoplakia of the lateral border of the right side of tongue in a 44-year-old HIV-seropositive female. The lesion was asymptomatic and microscopic examination of an incisional biopsy showed hyperkeratosis, mild acanthosis, and a mild, chronic inflammatory infiltrate. Special stains showed the presence of Epstein-Barr virus.

**Figure 2:**
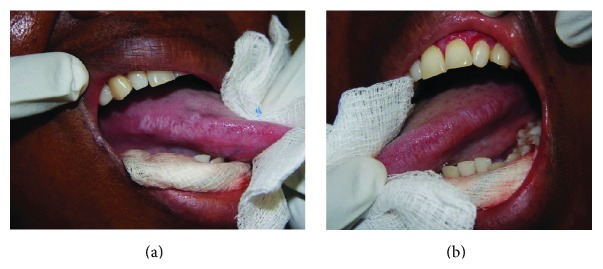
Mild oral hairy leukoplakia of the left and right lateral borders of the tongue in a 38-year-old HIV-seropositive female with a CD4+ T-cell count of 15 cells/mm^3^. The lesions were asymptomatic. The patient also had oral Kaposi sarcoma that was subsequently successfully treated with systemic cytotoxic chemotherapy.

Oral hairy leukoplakia is a benign, asymptomatic, white, hyperkeratotic lesion affecting primarily the lateral border of the tongue, unilaterally or bilaterally (Figures 1 and 2); but rarely it may occur elsewhere in the mouth. Its surface may be flat, vertically corrugated, or frankly hairy, and it affects severely immunocompromised subjects, most notably those infected with HIV [1–3].

**Figure 3:**
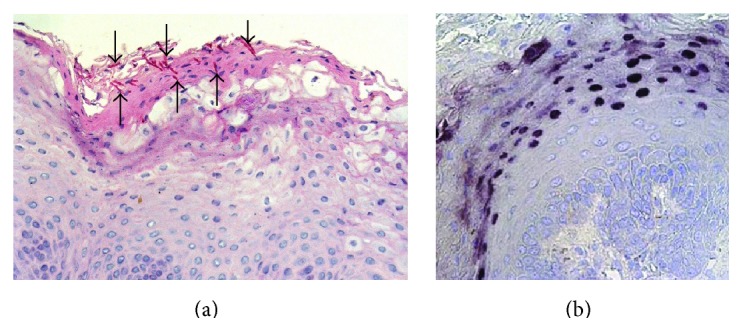
(a) Periodic acid-Schiff (PAS) stain showing several fungal hyphae embedded in the superficial keratin (arrows, magnification ×300); (b)* in situ* hybridization for EBV demonstrating numerous positive cells in the outer third of the epithelium (magnification ×300).

The characteristic microscopical features of oral hairy leukoplakia are epithelial hyperplasia, acanthosis, hyperkeratosis, and presence of koilocyte-like cells, but with little or no inflammatory cell infiltrate in the underlying lamina propria [1]. Candidal hyphae are not uncommonly present, but these represent a secondary fungal infection (Figure 3) [1].

There seems to be a causal link between Epstein-Barr virus (EBV) and oral hairy leukoplakia as EBV DNA, and EBV gene-encoded proteins are present in lesional cells. Oral hairy leukoplakia appears to be caused by productive replication of EBV in the oral mucosal epithelium, particularly of the lateral borders of the tongue [4–6]. In fact, in immunocompromised subjects, the oral epithelium supports both latent and productive EBV infections [2], with EBV replication and spread of virions occurring exclusively in terminally differentiated cells of the stratum spinosum and stratum granulosum [7]. Under these circumstances EBV is almost always present in the oral fluid [2, 8]. Another source of EBV in the oral fluid may be inflamed periodontal sites, as it has been shown that active periodontal pockets may harbour EBV DNA particles that contribute to the EBV load in the oral fluid [6].

EBV-induced oral hairy leukoplakia may be the first clinical manifestation of HIV infection, and in HIV-seropositive subjects it may be an indicator of progression to acquired immunodeficiency syndrome (AIDS). In HIV-seropositive subjects, a high HIV viral load and a low CD4+ T-cell count increase the risk of EBV-induced oral hairy leukoplakia [2–4].

In HIV-seropositive subjects, oral hairy leukoplakia is relatively asymptomatic and does not have any malignant potential, and as a rule it does not need treatment. About 10% of cases may improve spontaneously or may even resolve by improvement of the immune status after institution of highly active antiretroviral treatment (HAART). If for any reason treatment is to be undertaken, acyclovir, cryotherapy, laser treatment, surgical excision, or application of topical retinoids may ameliorate the lesion, but recurrence is not uncommon [4, 9–11].

## 2. General Characteristics of EBV

EBV is a member of the Herpesviridae family, subfamily Gammaherpesvirinae, and genus* Lymphocryptovirus* which infect B-lymphocytes and epithelial cells, but under certain circumstances also monocytes, Langerhans cells, and T-lymphocytes [7, 12]. There are two EBV genotypes, EBV-1 and EBV-2, that differ with regard to the DNA sequences of several genes, and with regard to their oncogenic potential [3, 13]. The prevalence of EBV-1 and EBV-2 infections differs according to geographic location: the frequency of EBV-1 is higher in Europe and in North America and of EBV-2 in Africa and in Oceania. In general, regardless of geographic location, HIV-seropositive subjects are frequently infected either by EBV-2, or by mixed EBV-1 and EBV-2, or by other genotypic variants which evolved by intrastrain recombination or by selective pressure-induced mutations [3, 10, 11, 13]. Homosexual men are more likely to be infected by EBV-2 and the frequency of infection correlates with the number of sexual partners [14].

The differences in geographical distribution of EBV-associated diseases, such as nasopharyngeal carcinoma in Southeast Asia, reflect evolution of genetic variants of the latent viral protein, LMP1, owing to positive selection pressures [15].

EBV-1 is potentially more oncogenic than EBV-2 and plays a role in the pathogenesis of several lymphoproliferative disorders and in nasopharyngeal carcinoma. However, very little is known about the differences in the pathogenic potential between EBV-1 and EBV-2 with respect to oral hairy leukoplakia, or how EBV drives its development [3]. Nevertheless, multiple EBV strains, interstrain and intrastrain recombinations, have been detected in the lesional cells of oral hairy leukoplakia [2, 13].

## 3. EBV Infection

Primary EBV infection usually occurs in childhood and is asymptomatic or mildly symptomatic, but when it occurs in adolescence or in adulthood it may cause infectious mononucleosis, a more significant condition. Following the primary infection, the virus persists lifelong in memory B-lymphocytes, and in fact the prevalence of latent EBV infection among adults worldwide is greater than 90% [4, 16]. EBV can replicate in B-lymphocytes and in oral squamous epithelial cells, and reactivation of latent EBV infection in these cells may result in productive infection [16] with the shedding of infectious EBV particles from oral epithelium into the oral fluid with the potential for viral transmission [17]. Thus, mature B-lymphocytes and oral keratinocytes serve as reservoirs of latent EBV infection [11].

Infectious EBV particles are found in the oral fluid of subjects with asymptomatic productive infection, who then have the capacity to spread the virus to uninfected subjects, or to subjects infected by different strains [6]. Although much less common, EBV infection can also be acquired from transplanted organs; and as EBV DNA particles are present in breast milk and in genital secretions, it is possible that EBV infection may also spread by breast feeding or by genital or orogenital sexual activity [4, 18]. However, Kusuhara et al. reported that breastfeeding is not a significant risk factor for EBV transmission [19].

Sexual transmission of EBV is also implied by the demonstration of EBV DNA in cervical secretions (8.9%) and in semen (16.8%) [20, 21]. Acquisition of EBV among young females correlates significantly with the number of sexual partners and is conclusively linked to their patterns of sexual behaviour [22].

In the oropharynx, EBV either infects primarily the oropharyngeal epithelium or passes through the mucosal epithelium and subsequently infects B-lymphocytes of the lymphoid-rich tissue of the tonsils and adenoids [4, 23]. There is a 30–50-day incubation period, whereafter replication and spread within the reticuloendothelial system occur [4]. The differentiation of B-lymphocytes into plasma cells stimulates productive replication of EBV with the ultimate release of EBV DNA particles that directly infect the oropharyngeal epithelial cells, where they may further replicate and spread [23]. However, while it is evident that oral keratinocytes of the lateral border of the tongue of HIV-seropositive subjects and other severely immunocompromised subjects support productive EBV replication, in HIV-seronegative immunocompetent subjects, the squamous cell epithelium of the tongue is only rarely supportive of productive EBV replication [8].

EBV infects epithelial cells by binding to the integrins, *α*v*β*6 and *α*v*β*8, on the surface of the plasma membrane of the target cells. This interaction triggers the fusion of EBV envelope with the cell membrane, transporting the virus into the cell. The mechanism by which EBV enters B-lymphocytes is different; but EBV continually shuttles between B-lymphocytes and epithelial cells, and this dynamic interaction promotes spreading and establishes persistence of the virus [11, 23]. It appears that, in EBV-associated carcinoma, transforming growth factor-*β*1 (TGF-*β*1) facilitates integrin assembly and cytoskeletal actin dynamics thus promoting EBV infection; and TGF-*β*1 also induces cell differentiation with the expression of the immediately early EBV gene, BZLF1, thus promoting productive replication of EBV [23].

EBV latent infection is characterized by expression of three EBV-encoded latency proteins (LMP 1–3) and six EBV nuclear antigens (EBNA 1–6). These latency proteins play important roles in EBV-mediated transformation of infected mature B-lymphocytes [24]. However, it has become clear that productive EBV replication and environmental factors also play important roles in the pathogenesis of EBV-associated diseases. EBV replication increases the reservoir of latently infected cells, while environmental factors influence the complex interactions between EBV and the host with the capacity to modulate the expression of EBV latent oncogenes [24].

In latent EBV infection, the double-stranded viral DNA genome is replicated once in every cell cycle [25]; but not all the factors which determine whether EBV infection remains latent or reactivates to become productive are known. However, the fitness of the immune system, cellular stress signals related to hypoxia or to DNA damage, and cytokines in the local microenvironment all play roles in EBV reactivation [25].

In epithelial cells, on the one hand productive EBV replication is induced by the cellular genetic factor B-lymphocyte-induced maturation protein 1 (Blimp1) expressed during the process of terminal differentiation, while, on the other hand, perturbed cellular differentiation allows latent but not productive EBV infection. In fact, an isoform of p63 protein (ΔNp63), a member of the p53 family, is normally expressed in undifferentiated epithelial basal cells. However, in EBV-associated epithelial malignancies it is overexpressed, contributing to the persistence of undifferentiated keratinocytes expressing latent EBV genes that play an important role in the pathogenesis of EBV-associated carcinoma. Thus, cellular factors associated with cell differentiation will favour productive EBV infection, while maintenance of an undifferentiated cellular state will be associated with latent EBV infection [23].

During productive EBV infection, there is an orderly sequential expression of immediately early and early genes, followed by viral DNA replication and then by expression of late viral genes and their encoded proteins [6, 8, 9]. Of these many genes, the immediately early gene, BZFL1, is necessary and sufficient for activation of the productive EBV replication cascade [16], and it thus plays an important role in the pathogenesis of oral hairy leukoplakia [23].

EBV proteins in the local microenvironment have the capacity, directly or indirectly, to induce inflammatory responses, to activate intracellular signalling pathways related to cell cycle progression, and to mediate immune evasion [6, 24], thus favouring the development of EBV-related conditions, including benign and malignant neoplasms, and oral hairy leukoplakia.

## 4. EBV-Associated Immune Response and Immune Evasion

Despite the inflammatory and potentially oncogenic nature of EBV, in most of EBV-infected subjects effective host immune responses prevent the development of EBV-related diseases; but immunocompromised subjects are at risk [23]. The dynamics of latent EBV infection, subclinically active infection, or the frank development of EBV-associated diseases is thus determined by the capacity of the immune system to suppress the infection [23].

Early in the course of primary EBV infection, elements of the innate immune system such as cytokines, natural killer cells, and immune dendritic cells play important roles in controlling the infection. Subsequently adaptive EBV-specific cytotoxic T-lymphocytes become the predominant agents of the immune response [6].

EBV activates toll-like receptors of keratinocytes and monocytes, stimulating the production of proinflammatory cytokines that mediate protective immunoinflammatory responses with the capacity to limit the spread of productive EBV infection and to control reactivation of latent infection. However, as a counter to these immunoinflammatory responses, EBV can activate a variety of mechanisms mediating immune evasion, thus ensuring its own persistence in mature B-lymphocytes and epithelial cells, promoting the later development of EBV-associated inflammatory and neoplastic diseases. Latent membrane protein 1-derived peptides (LMP1) from EBV-positive tumour cells can inhibit T-lymphocyte proliferation, natural killer-mediated cytotoxicity, and EBV-specific production of interferon gamma, with the generation of an immunosuppressive microenvironment [26]. EBV-infected granulocytes, monocytes, macrophages, and immune dendritic cells in the local microenvironment are functionally impaired, and they generate a dysregulated cytokine network with reduction in both the number and the function of cytotoxic CD8+ T-lymphocytes [24].

In HIV-associated oral hairy leukoplakia, there is a decrease in the number of Langerhans cells in the affected epithelium with consequent downregulation of EBV-specific cytotoxic CD8+ T-lymphocyte responses that would otherwise inhibit productive EBV replication [10, 27, 28]. This local immune suppression may explain the very few immunoinflammatory cells in the lamina propria of oral hairy leukoplakia [28]. Subjects with a high HIV viral load and a low CD4+ T-cell count are at increased risk of HIV-associated oral hairy leukoplakia.

## 5. EBV-Associated Oral Hairy Leukoplakia

Certain mechanisms have been proposed to explain infection of the oral epithelium by EBV [2, 10, 11, 18]. Firstly, latent EBV in circulating B-lymphocytes may undergo reactivation, producing and releasing virions which may infect circulating monocytes. Subsequently, those blood-borne EBV-infected monocytes that enter the lamina propria of the oral mucosa can differentiate into latently infected macrophages, and into Langerhans cell precursors which then migrate into the oral epithelium. The mature latently infected Langerhans cells reside in the basal/parabasal layer of the oral epithelium, and if EBV becomes reactivated, the virus will be transferred through the dendritic processes of Langerhans cells to keratinocytes in the stratum spinosum and stratum granulosum where productive EBV replication may ensue [7, 18]. Similarly, episodes of productive EBV replication can also occur in B-lymphocytes within the oral lymphoid tissue with the release of virions which will infect nearby monocytes, macrophages, and Langerhans cell precursors which then, as described above, migrate into and infect the oral epithelium [7, 10, 11].

In some cases, the epithelial cell cycle may be dysregulated by this productive EBV replication resulting in the clinical manifestation of oral hairy leukoplakia. However, in fact in HIV-associated oral hairy leukoplakia there is a decrease in the number of oral Langerhans cells, perhaps because they either are killed by antigen specific cytotoxic CD8+ T-cells or migrate from the epithelium [7, 10].

A second mechanism of EBV infection of the oral epithelium can be that virions that shed into the oral fluid from productive EBV replication in the hosts' oral/oropharyngeal lymphoid tissue or epithelium will penetrate intercellular microdefects in the oral epithelial surface where the virus can invade terminally differentiated keratinocytes and induce their proliferation resulting in oral hairy leukoplakia [10, 11, 18].

The immediate-early gene BZLF1 is necessary to bring about the intracellular switch from latent to productive infection. In oral hairy leukoplakia the BZLF1 is restricted to cells of the stratum spinosum and stratum granulosum which express the Blimp1 that acts as a transcription factor in the terminal differentiation of keratinocytes [16]. Since neither Blimp1 nor BZLF1 can be detected in the basal cell layer of the oral epithelium it is reasonable to assume that basal cells are not involved in productive EBV infection in oral hairy leukoplakia [16, 23].

A third possible mechanism leading to dysregulation of the cell cycle of terminally differentiated oral keratinocytes resulting in oral hairy leukoplakia may be that latent EBV becomes reactivated as infected cells of the lower strata of epithelium rise to the surface of the epithelium, with viral replication and spread occurring in these layers [2].

The intracellular molecular mechanisms that determine the pattern of epithelial hyperplasia that give rise to the clinical and microscopical features of oral hairy leukoplakia are not clear. However, it has been proposed that some latent EBV proteins (e.g., LMP1) can stimulate intracellular signalling pathways with the downstream activation of transcription factors NF-*κ*B and AP1, with consequent upregulation of expression of genes inducing cell proliferation and promoting cell survival [29]. Normally, the keratinocytes in the stratum spinosum and stratum granulosum are postmitotic suprabasal terminally differentiated cells, which have progressed through the sequence of maturation from the basal and suprabasal layers of the epithelium and are on their way to exfoliate at the surface [2]. However, in oral hairy leukoplakia EBV seems to interfere with the normal differentiation process of keratinocytes in the suprabasal layers of the oral epithelium, resulting in abnormal persistence of the capacity for cell division of EBV-infected keratinocytes. The outcome of this drives the pathological process of epithelial hyperplasia, acanthosis, and hyperkeratosis that are characteristic to oral hairy leukoplakia [29].

## 6. Conclusion

Although it is rare in immunocompetent subjects, in a small minority of immunocompromised subjects, particularly in those who are HIV-seropositive, productive EBV infection of the oral epithelium will result in oral hairy leukoplakia. It is clear that oral EBV infection of itself is insufficient to cause oral hairy leukoplakia and therefore other hosts, EBV-specific, and perhaps environmental factors must play roles in the pathogenesis of oral hairy leukoplakia.

## References

[1] Braz-Silva P. H., de Rezende N. P. M., Ortega K. L., de Macedo Santos R. T., de Magalhães M. H. C. G. (2008). Detection of the Epstein-Barr virus (EBV) by in situ hybridization as definitive diagnosis of hairy leukoplakia. *Head and Neck Pathology*.

[2] Hille J. J., Webster-Cyriaque J., Palefski J. M., Raab-Traub N. (2002). Mechanisms of expression of HHV8, EBV and HPV in selected HIV-associated oral lesions. *Oral Diseases*.

[3] Robaina T. F., Valladares C. P., Tavares D. S. (2008). Polymerase chain reaction genotyping of Epstein-Barr virus in scraping samples of the tongue lateral border in HIV-1 seropositive patients. *Memorias do Instituto Oswaldo Cruz*.

[4] Di Lernia V., Mansouri Y. (2013). Epstein-Barr virus and skin manifestations in childhood. *International Journal of Dermatology*.

[5] González X., Correnti M., Rivera H., Perrone M. (2010). Epstein Barr Virus detection and latent membrane protein 1 in oral hairy leukoplakia in HIV+ Venezuelan patients. *Medicina Oral, Patologia Oral y Cirugia Bucal*.

[6] Slots J., Saygun I., Sabeti M., Kubar A. (2006). Epstein-Barr virus in oral diseases. *Journal of Periodontal Research*.

[7] Tugizov S., Herrera R., Veluppillai P., Greenspan J., Greenspan D., Palefsky J. M. (2007). Epstein-Barr virus (EBV)-infected monocytes facilitate dissemination of EBV within the oral mucosal epithelium. *Journal of Virology*.

[8] Herrmann K., Frangou P., Middeldorp J., Niedobitek G. (2002). Epstein-Barr virus replication in tongue epithelial cells. *Journal of General Virology*.

[9] Walling D. M., Flaitz C. M., Nichols C. M. (2003). Epstein-Barr virus replication in oral hairy leukoplakia: response, persistence, and resistance to treatment with valacyclovir. *Journal of Infectious Diseases*.

[10] Walling D. M., Ling P. D., Gordadze A. V., Montes-Walters M., Flaitz C. M., Nichols C. M. (2004). Expression of epstein-barr virus latent genes in oral epithelium: determinants of the pathogenesis of oral hairy leukoplakia. *Journal of Infectious Diseases*.

[11] Walling D. M., Etienne W., Ray A. J., Flaitz C. M., Nichols C. M. (2004). Persistence and transition of Epstein-Barr virus genotypes in the pathogenesis of oral hairy leukoplakia. *Journal of Infectious Diseases*.

[12] Grywalska E., Rolinski J. (2015). Epstein-barr virus–associated lymphomas. *Seminars in Oncology*.

[13] Webster-Cyriaque J., Raab-Traub N. (1998). Transcription of Epstein-Barr virus latent cycle genes in oral hairy leukoplakia. *Virology*.

[14] van Baarle D., Hovenkamp E., Dukers N. H. T. M. (2000). High prevalence of Epstein-Barr virus type 2 among homosexual men is caused by sexual transmission. *Journal of Infectious Diseases*.

[15] Burrows J. M., Bromham L., Woolfit M. (2004). Selection pressure-driven evolution of the Epstein-Barr virus-encoded oncogene LMP1 in virus isolates from Southeast Asia. *Journal of Virology*.

[16] Buettner M., Lang A., Tudor C. S. (2012). Lytic Epstein-Barr virus infection in epithelial cells but not in b-lymphocytes is dependent on blimp. *Journal of General Virology*.

[17] Tsao S. W., Tsang C. M., Pang P. S., Zhang G., Chen H., Lo K. W. (2012). The biology of EBV infection in human epithelial cells. *Seminars in Cancer Biology*.

[18] Walling D. M., Ray A. J., Nichols J. E., Flaitz C. M., Nichols C. M. (2007). Epstein-Barr virus infection of Langerhans cell precursors as a mechanism of oral epithelial entry, persistence, and reactivation. *Journal of Virology*.

[19] Kusuhara K., Takabayashi A., Ueda K. (1997). Breast milk is not a significant source for early Epstein-Barr virus or human herpesvirus 6 infection in infants: a seroepidemiologic study in 2 endemic areas of human T-cell lymphotropic virus type I in Japan. *Microbiology and Immunology*.

[20] Enbom M., Strand A., Falk K. I., Linde A. (2001). Detection of epstein-barr virus, but not human herpesvirus 8, DNA in cervical secretions from Swedish women by real-time polymerase chain reaction. *Sexually Transmitted Diseases*.

[21] Kapranos N., Petrakou E., Anastasiadou C., Kotronias D. (2003). Detection of herpes simplex virus, cytomegalovirus, and Epstein-Barr virus in the semen of men attending an infertility clinic. *Fertility and Sterility*.

[22] Woodman C. B. J., Collins S. I., Vavrusova N. (2005). Role of sexual behavior in the acquisition of asymptomatic Epstein-Barr virus infection: a longitudinal study. *Pediatric Infectious Disease Journal*.

[23] Tsao S.-W., Tsang C. M., To K.-F., Lo K.-W. (2014). The role of Epstein-Barr virus in epithelial malignancies. *Journal of Pathology*.

[24] Dolcetti R. (2015). Cross-talk between Epstein-Barr virus and microenvironment in the pathogenesis of lymphomas. *Seminars in Cancer Biology*.

[25] Kenney S. C., Mertz J. E. (2014). Regulation of the latent-lytic switch in Epstein-Barr virus. *Seminars in Cancer Biology*.

[26] Dukers D. F., Meij P., Vervoort M. B. H. J. (2000). Direct immunosuppressive effects of EBV-encoded latent membrane protein 1. *The Journal of Immunology*.

[27] Lilly E. A., Cameron J. E., Shetty K. V. (2005). Lack of evidence for local immune activity in oral hairy leukoplakia and oral wart lesions. *Oral Microbiology and Immunology*.

[28] Hahn A. M., Huye L. E., Ning S., Webster-Cyriaque J., Pagano J. S. (2005). Interferon regulatory factor 7 is negatively regulated by the Epstein-Barr virus immediate-early gene, BZLF-1. *Journal of Virology*.

[29] Webster-Cyriaque J., Middeldorp J., Raab-Traub N. (2000). Hairy leukoplakia: An unusual combination of transforming and permissive Epstein-Barr virus infections. *Journal of Virology*.

